# Targeting SARS-CoV-2 nonstructural protein 15 endoribonuclease: an *in silico* perspective

**DOI:** 10.2217/fvl-2020-0233

**Published:** 2021-07-13

**Authors:** Shafi Mahmud, Abdo A Elfiky, Al Amin, Sumon Chandro Mohanto, Ekhtiar Rahman, Uzzal Kumar Acharjee, Abu Saleh

**Affiliations:** 1 ^1^Genetic Engineering & Biotechnology, University of Rajshahi, Rajshahi, 6205, Bangladesh; 2 ^2^Biophysics Department, Faculty of Science, Cairo University, Giza, Egypt; 3 ^3^Institute of Biological Science, University of Rajshahi, Rajshahi, 6205, Bangladesh

**Keywords:** COVID-19, endoribonuclease, molecular docking, Nsp15, SARS-CoV-2

## Abstract

The newly emerged human coronavirus, SARS-CoV-2, had begun to spread last year and sparked worldwide. In this study, molecular docking is utilized to test some previously approved drugs against the SARS-CoV-2 nonstructural protein 15 (Nsp15). We screened 23 drugs, from which three (saquinavir, valrubicin and aprepitant) show a paramount predicted binding affinity (-9.1, -9.6 and -9.2 kcal/mol, respectively) against SARS-CoV-2 Nsp15. Moreover, saquinavir and aprepitant make nonbonded interactions with Leu201 in the active site cavity of Nsp15, while the drug valrubicin interacts with Arg199 and Leu201. This binding pattern may be effective against the targeted protein, leading to Nsp15 blockage and virus abolition. Additionally, the pharmacological properties of the screened drugs are known since they have been approved against different viruses.

## Graphical abstract

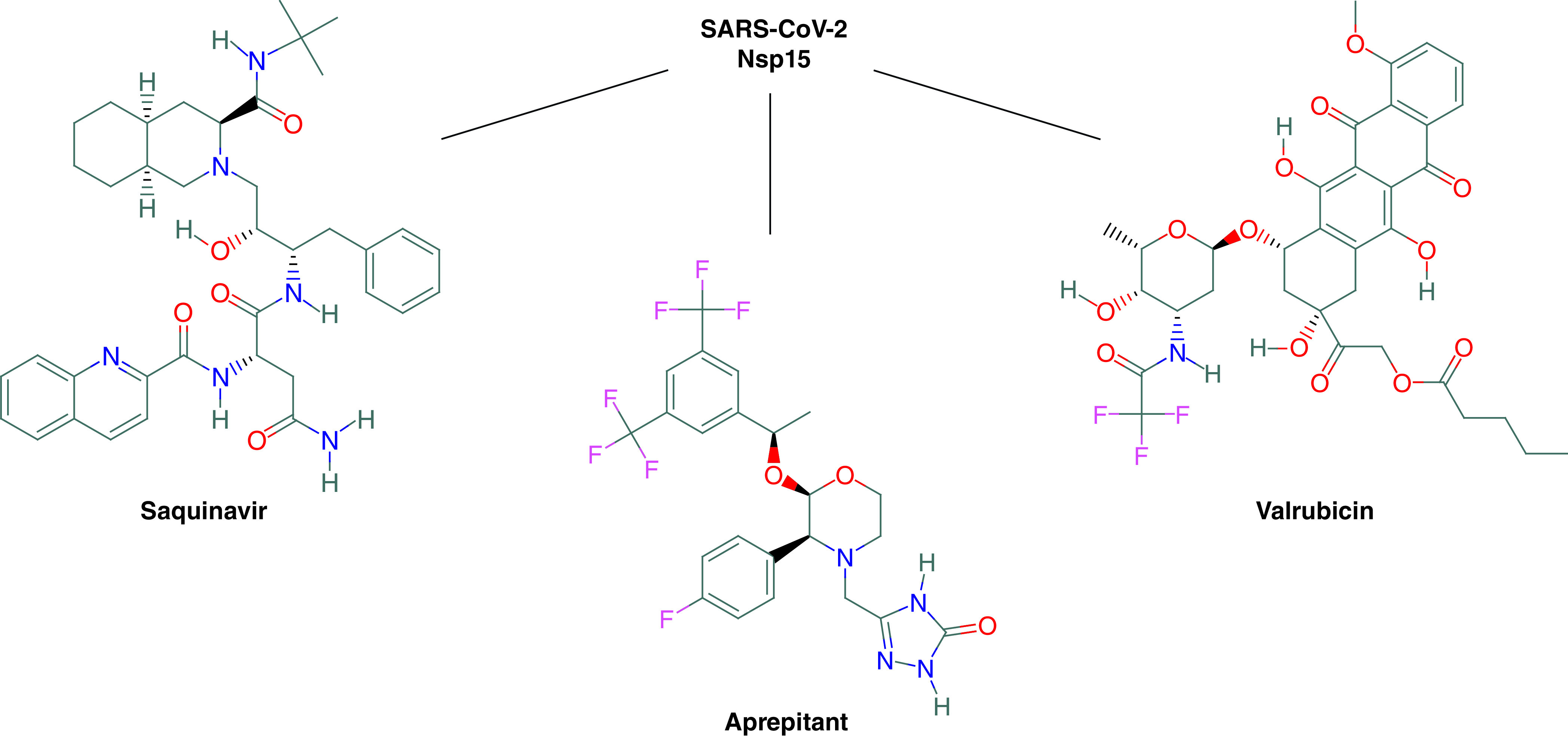

In December 2019, a novel coronavirus, SARS-CoV-2, sparked in Wuhan, China, and became a pandemic in March 2020 [1]. Almost 142 million people are infected, with more than 3 million reported deaths. The common symptoms of this disease include cough, fever, shortness of breath, fatigue, loss of taste and muscle and abdominal pain. The timeframe from exposure to the appearance of symptoms can generally be around 5 days but may range from 2 to 14 days [2,3].

The whole RNA genome of SARS-CoV-2 is 28–32 kb long. It encodes; four structural proteins (spike [S], matrix [M], envelope [E] and nucleocapsid [N]), open reading frame (ORF), nonstructural polyprotein and five accessory proteins (ORF3a, ORF6, ORF7, ORF8, ORF9) required for the function and assembly of SARS-CoV-2 [4–6]. The replicase gene of this virus encodes two ORFs and is translated into pp1a and pp1ab. The viral proteases (papain-like protease and 3C-like protease) are processed from these polypeptides [6,7]. It helps to hide viral RNA from host defense (RNA degradation) [8]. Additionally, the nonstructural protein 15 (Nsp15) has endoribonuclease activity and reported interfering with IFN-β production and associated with retinoblastoma tumor suppressor protein in coronaviruses [9–11]. Nsp15 has endoribonuclease activity as it cleaves RNA at the 3′ of uridylates, followed by the formation of 2′-3′ cyclic products, just like the RNase A activity [12]. Targeting such essential viral protein could interfere with the SARS-CoV-2 life cycle [13].

Several bioactive compounds (phytochemicals) were previously tested *in silico* against Nsp15 of SARS-CoV-2, including asparoside-C, asparoside-F, asparoside-D, rutin and racemoside-A [14]. These compounds have predicted binding affinities greater than -7.17 kcal/mol. On the other hand, glyasperin A, isoliquiritinapioside, among other phytochemicals from *Glycyrrhiza* *glabra*, show lower (better) predicted binding affinities (down to -9.2 kcal/mol) against SARS-CoV-2 Nsp15 [15]. The safety profiles of these phytochemical-based compounds are missing, while other studies tested two US FDA-approved drugs (of known safety profiles) against the SARS-CoV-2 Nsp15, namely glisoxepide and idarubicin [16]. These two drugs show stronger binding against Nsp15 compared with the physiological uridine triphosphate [16].

We are trying in the current study to test different groups of successfully approved drugs, in other viruses, against SARS-CoV-2 Nsp15. A conventional antipsychotic drug, perphenazine, works by decreasing abnormal excitement in the brain. It is frequently used in the US to treat schizophrenia to control severe nausea and vomiting in adults [17]. On the other hand, ribavirin is a synthetic guanosine nucleoside used for its broad-spectrum activity against several RNA and DNA viruses [18]. Remdesivir is a promising antiviral drug against various RNA viruses, including the Ebola virus, SARS-CoV and Middle East respiratory syndrome (MERS) coronavirus [19–21]. At the same time, it was recently approved by the FDA for the treatment of the Ebola virus and SARS-CoV-2 (emergency use only approval) [22,23]. Additionally, epirubicin is used in chemotherapy as an active antineoplastic agent against malignancies such as breast cancer, lymphoma, lung cancer, ovarian, liver cancers and sarcomas [24].

Moreover, amprenavir, lopinavir, fosamprenavir, atazanavir, saquinavir, indinavir and tipranavir are HIV protease inhibitors. Among these drugs, amprenavir has received marketing approval from the FDA, while fosamprenavir (nucleoside reverse transcriptase inhibitors) has been approved in more than 40 countries. In contrast, nelfinavir has been used since the mid-90s against HIV [25–29]. Moreover, vapreotida has anticancer activity and is being used in photothermal therapy [30]. Bepotastine has been approved in Japan as a second-generation antihistamine for the treatment of allergic rhinitis [31]. Recently, lopinavir, along with ritonavir, has already been used as a trial at Jin Yin-Tan Hospital (Wuhan, China) to give comfort to hospitalized adult patients affected severely with COVID-19 [32,33]. Moreover, galidesivir has broad-spectrum antiviral activity against many RNA virus families like bunyaviruses, arenaviruses, paramyxoviruses, coronaviruses, flaviviruses and phleboviruses [34].

In this study, chemically active and structurally diverse drugs are selected and tested for their possible inhibitory effect against SARS-CoV-2 Nsp15 using molecular modeling. The *in silico* methods such as molecular docking and dynamics simulations are successful in finding viable solutions for emerging infections [35–40]. Computational methods help reduce the time, effort and money in finding a solution in the drug design, especially with such pandemic infectious diseases [41,42].

## Materials & methods

### Ligands & protein preparations

The chemical compounds are downloaded from the PubChem database [43] as a structure data file format then subjected to geometry optimization. The Merck molecular force field 94 is employed through the steepest gradient descent algorithm with 2000 minimization steps [44,45]. Twenty-three previously approved drugs are selected based on their potential inhibitory activity against different viruses (see Supplementary Table 1). They include anti-HIV, anti-SARS-CoV, anti-MERS coronavirus, anti-HCV, anti-Ebola virus and other approved medicines. On the other hand, the x-ray diffraction solved structure of SARS-CoV-2 Nsp15 endoribonuclease (Protein Data Bank [PDB] ID: 6W01) was downloaded from the PDB database (1.9 Å resolution) [46]. The water molecules and heteroatoms are removed using Discovery Studio software [47]. Finally, the protein structure was minimized in the Swiss PDB Viewer software [48] with the help of the GROMOS 43B1 force field [49].

### Molecular docking

The molecular docking study is employed to test and predict the possible binding pattern of the drugs against SARS-CoV-2 Nsp15 endoribonuclease. The docking method is validated through docking the citrate compound into the Nsp15 structure (redocking) PDB ID: 6W01 chain A. Supplementary Figure 1 shows how the docked citrate molecule coincides with the citrate found in the solved structure. Additionally, the docking score (-5.1 kcal/mol) was in agreement with previous work [50]. After that, both the protein and the ligand files were converted into PDBQT format, the acceptable file format in AutoDock Vina (Scripps Research, CA, USA), after the addition of any missed hydrogen atoms and charges (Kollman and Gasteiger) utilizing AutoDock Tools software [51,52]. The center of the grid box was set at (-54.9, 51.0, 24.0 Å), while the box dimensions were set to be large enough to cover the entire protein active site region (62.3 × 72.0 × 59.7 Å^3^). This box covers the entire Nsp15 active site residues (HIS235, HIS250, LYS290, SER294, THR341 and TYR343) to check for the possible binding sites with the tested ligands [10]. Finally, the drug protein–ligand complexes are screened based on the calculated binding energies and subjected to analysis using Discovery Studio (Dassault Systèmes, Vélizy-Villacoublay, France). At the same time, PyMOL (Schrödinger, Inc., NY, USA) was utilized as additional software to visualize the complexes [53].

## Results

**Figure 1.**
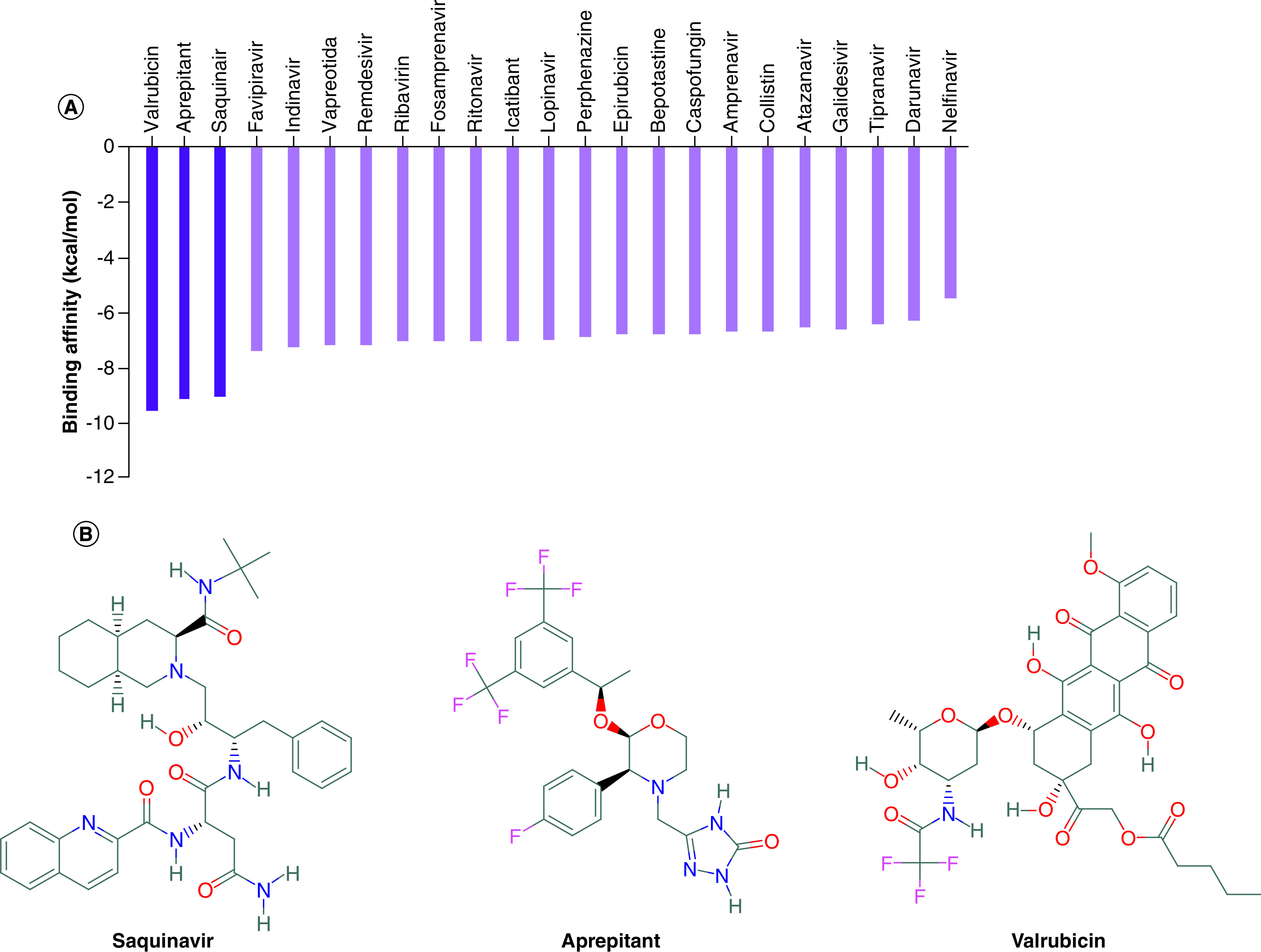
Molecular docking of the compounds to the nonstructural protein 15. Molecular docking **(A)** The binding affinities (in kcal/mol) calculated for the selected drugs against SARS-CoV-2 nonstructural protein 15 using AutoDock Vina software. **(B)** The 2D structures of the best three compounds (valrubicin, aprepitant and saquinavir), based on the binding affinity.

Molecular docking was performed using AutoDock Vina software to test and rank the 23 candidate drugs against SARS-CoV-2 Nsp15. As shown in Figure 1A, the binding affinities range from -5.5 kcal/mol (nelfinavir) down to -9.6 kcal/mol (valrubicin). The drugs ranked by its binding affinity against SARS-CoV-2 Nsp15 are: valrubicin, aprepitant, saquinavir, favipiravir, indinavir, vapreotida, remdesivir, ribavirin, fosamprenavir, ritonavir, icatibant, lopinavir, perphenazine, epirubicin, bepotastine, caspofungin, amprenavir, colistin, atazanavir, galidesivir, tipranavir, darunavir and nelfinavir. The drugs valrubicin, aprepitant and saquinair show excellent binding affinities to SARS-CoV-2 Nsp15 (-9.1, -9.6 and -9.2 kcal/mol, respectively) as shown in Figure 1A. The 2D structures of the best three compounds are shown in Figure 1B.

**Figure 2.**
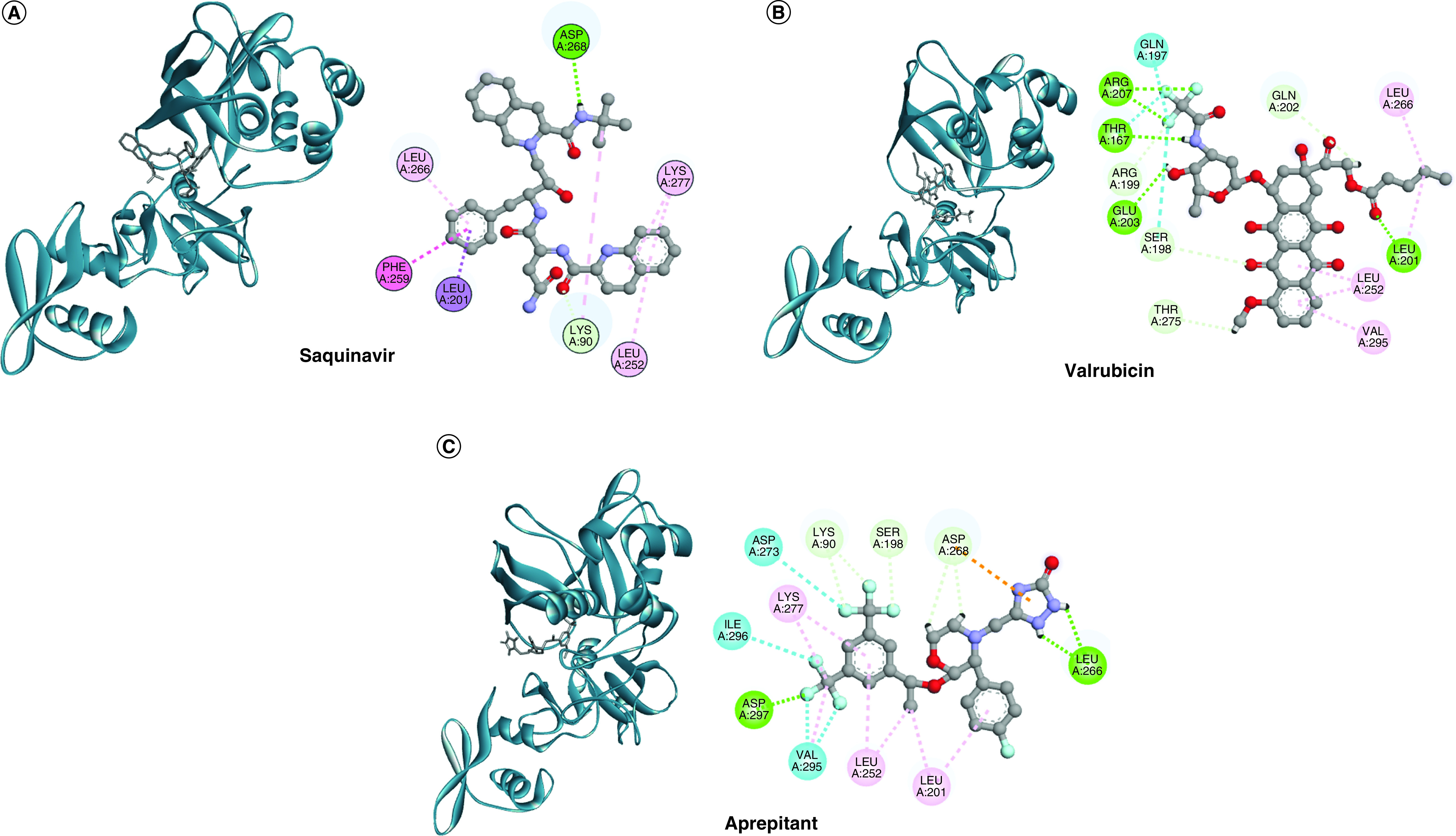
Post-docking analysis. The 3D structures (left) and the 2D interaction scheme (right) of the complexes formed upon docking the drugs to SARS-CoV-2 nonstructural protein 15. **(A)** Saquinavir. **(B)** Valrubicin. **(C)** Aprepitant. A variety of bonded patterns, including hydrogen (green), halogen bond (cyan), alkyl (pink), π-alkyl (violet), and π-π-T-shaped (red) bonds are depicted as dashed colored lines on the three complexes. The amino acids are colored according to the interaction involved.

By analyzing the docking complexes, saquinavir formed two hydrogen bonds with Lys90 and Asp268 of the SARS-CoV-2 Nsp15. Additionally, one π-sigma bond with Leu201, one π-π-T shaped with Phe259, one Alkyl with Lys277 and two π-alkyl (Leu252 and Leu266) are established in the saquinavir-SARS-CoV-2 Nsp15 complex (Figure 2). On the other hand, valrubicin shows more nonbonded interaction with the endoribonuclease compared with saquinavir (Figure 2). The valrubicin-SARS-CoV-2 Nsp15 complex shows eight hydrogen bonds (Thr167, Ser198, Arg199, Leu201, Gln202, Glu203, Arg207 and Thr275), two π-alkyl bonds (Leu252 and Val295) and one alkyl bond (Leu266). Interestingly, three more halogen bonds are also formed (Thr167, Gln197 and Ser198) for this complex, which was absent for the saquinavir-SARS-CoV-2 Nsp15 complex.

Table 1 lists the formed interactions established upon docking the best three drugs (saquinavir, valrubicin and aprepitant) into SARS-CoV-2 Nsp15 active site. Additionally, the bond distances are listed between the brackets to indicate the bond strength.

**Table 1.** Interactions established upon docking the drugs saquinair, valrubicin and aprepitant into the active site of SARS-CoV-2 nonstructural protein 15.

Drug name	PubChem CID	Hydrogen bond (distance Å)	Hydrophobic interaction (distance Å)	Halogen (distance Å)
Saquinavir	441243	Asp268 (2.20) Lys90 (2.74)	π-sigma Leu201 (2.66) π-π-T-shaped Phe259 (5.47) Alkyl Lys277 (4.16) π-alkyl Leu252 (5.39) Leu266 (5.31)	-
Valrubicin	454216	Glu203 (2.59) Thr167 (2.25) Leu201 (2.37) Arg207 (2.21) Gln202 (2.86) Thr275 (2.85) Ser198 (2.77) Arg199 (2.66)	Alkyl Leu266 (4.68) π-alkyl Leu252 (5.28) Val295 (5.38)	Thr167 (3.17) Gln197 (3.52) Ser198 (3.05)
Aprepitant	135413536	Leu266 (1.92) Asp297 (2.86) Asp268 (2.90) Lys90 (2.77) Ser198 (2.40)	Alkyl Leu201 (3.94) Leu252 (4.76) Lys277 (4.75) Val 295 (4.60)	Asp273 (3.69) Val295 (3.12) Ile296 (3.93)

Docked complexes are analyzed using Discovery Studio and also by PyMOL software.

## Discussion

Molecular docking has become the most widely used approach and has substantial success stories during pandemics [54,55]. Docking, combined with other computational methods, may play an essential role in determining drug–protein relationships and their binding pattern [56,57].

It was reported that the π-alkyl bonds contribute well to better binding, which is responsible for the more stable and more durable binding in biological systems [58,59]. Since valrubicin and saquinavir both contribute several π-alkyl interactions with Nsp15, it may play a crucial role in improved affinity compared with other drugs. Similarly, the aprepitant-SARS-CoV-2 Nsp15 complex is stabilized by five hydrogen bonds (Lys90, Ser198, Leu266, Asp268 and Asp297) and four alkyl bonds (Leu201, Leu252, Lys277 and Val295). Like valrubicin, aprepitant also forms halogen contacts with Nsp15 but at different positions (Asp273, Val295 and Ile296). These halogen bonds contribute to the binding affinity with the macromolecular systems [60]. The screened drugs, valrubicin and aprepitant, provide more hydrogen bonds with SARS-CoV-2 Nsp15, where this bonding pattern impacts the overall complex stability and molecular recognition of the system [61].

Some of the interacting residues in Figure 2 are surface accessible (gray shadowed circles) that reflect its surface exposed property. These residues are LEU266 in the three drugs, LYS90 and ASP268 in saquinavir and aprepitant and LEU201 in saquinavir and valrubicin. Valrubicin has more surface accessible residues such as S198, ARG199, GLN202 and GLU203. These residues lie near the active site cavity of the Nsp15 (HIS235, HIS250, LYS290, SER294, THR341 and TYR343)

To be considered a possible drug candidate, the drug molecules have to maintain specific parameters, although violations can sometimes be present [62]. The tested drugs are already approved and hence its toxicity and their ADMET properties are known. Additionally, the drug saquinavir has been reported as a potential SARS-CoV-2 RdRp main protease (Mpro) inhibitor [63]. It was tested through docking to the dimeric Mpro and then molecular dynamics simulation followed by molecular mechanics/generalized Born surface area calculations [64]. Saquinavir was the best among 12 FDA-approved drugs in the study and was suggested as a potential SARS-CoV-2 Mpro inhibitor [63].

In summary, the three approved drugs, saquinavir, valrubicin and aprepitant, may be potential candidates that can bind to SARS-CoV-2 Nsp15 protein effectively with excellent *in silico* binding affinity values. Moreover, the binding of these three drug molecules in the active sites of Nsp15 may contradict viral infection. However, further in-depth simulation studies (dynamics) and experimental validation is suggested as future work. Additionally, optimization of these drugs may help in finding a better cure against SARS-CoV-2.

## Conclusion

The WHO declared COVID-19 as a pandemic in March 2020. This study tested the binding affinity and mode of 23 clinically trialed, approved (FDA) drugs to repurpose it against SARS-CoV-2 Nsp15. Valrubicin, saquinavir and aprepitant showed promising results in binding the viral protein Nsp15, among other drugs. Besides, saquinavir was suggested as a potential inhibitor of SARS-CoV-2 Mpro as well in a previous study. So, it can be a double-target medicine that could inhibit SARS-CoV-2 infectivity. This needs more in-depth simulation work and experimental validation studies before the clinical trials. Moreover, the efficacy and reactivity of the drugs could be improved by modifying them to fit the SARS-CoV-2 Nsp15 active site pocket precisely.

**Executive summary**

- US FDA-approved drugs can target SARS-CoV-2 nonstructural protein 15 (Nsp15).
- Molecular docking reveals that valrubicin, aprepitant and saquinavir are strongly associated with Nsp15 of SARS-CoV-2.
- Both H-bonds and halogen bonds are the driving force for the binding in the case of valrubicin and aprepitant, while few hydrophobic contacts are present.
- Saquinavir forms mainly hydrophobic contacts with Nsp15 and could be a double blocker targeting both Nsp15 and Mpro of SARS-CoV-2.
