# Supplementary material for: Targeting SARS-CoV-2 nonstructural protein 15 endoribonuclease: an in silico perspective
Source: Future Virol. 2021 Jul 13:10.2217/fvl-2020-0233. doi: 10.2217/fvl-2020-0233 (PMC8285111; doi:10.2217/fvl-2020-0233)

Table S1: Binding affinity of the drug molecules against SARS-CoV-2 Nsp15.

| Compound Name | PubChem CID | Docking Energy (kcal/mol) |
| --- | --- | --- |
| Perphenazine | 4748 | -6.9 |
| Ribavirin | 37542 | -7.1 |
| Epirubicin | 41867 | -6.8 |
| Nelfinavir | 64143 | -5.5 |
| Amprenavir | 65016 | -6.7 |
| Vapreotida | 71306 | -7.2 |
| Lopinavir | 92727 | -7.0 |
| Fosamprenavir | 131536 | -7.1 |
| Atazanavir | 148192 | -6.6 |
| Bepotastine | 164522 | -6.8 |
| Darunavir | 213029 | -6.3 |
| Ritonavir | 392622 | -7.1 |
| Saquinair | 441243 | -9.1 |
| Valrubicin | 454216 | -9.6 |
| Favipiravir | 492405 | -7.4 |
| Caspofungin | 2826718 | -6.8 |
| Collistin | 5311054 | -6.7 |
| Indinavir | 5362440 | -7.3 |
| Icatibant | 6918173 | -7.1 |
| Galidesivir | 10445549 | -6.6 |
| Tipranavir | 54682461 | -6.4 |
| Remdesivir | 121304016 | -7.2 |
| Aprepitant | 135413536 | -9.2 |
|  |  |  |

Supplementary Figure:
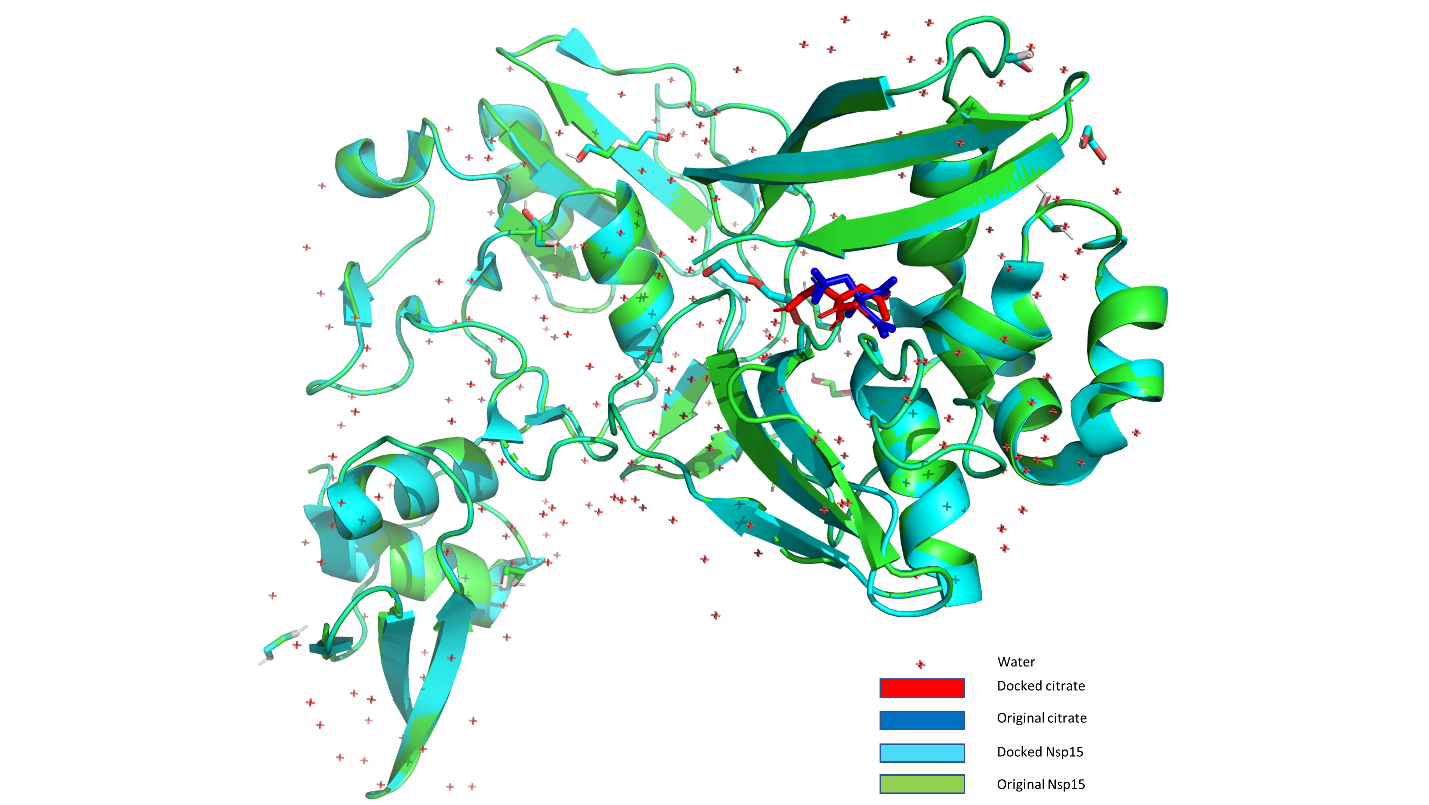

Supplement: Supplementary file 1 [file Supplementary_Data.docx]
